# Effect of Probiotic and Synbiotic Supplementation on Growth Performance, Serum Parameters and Gut Histomorphometry in Broiler Chickens Challenged With *Eimeria*

**DOI:** 10.1002/vms3.70842

**Published:** 2026-02-07

**Authors:** Subash Chhetri, Dinesh Kumar Singh, Bibash Bahadur Tiwari, Sushil Neupane, Bikas Raj Shah, Deepak Subedi

**Affiliations:** ^1^ Ministry of Agriculture and Livestock Development Kathmandu Nepal; ^2^ Institute of Agriculture and Animal Science Tribhuvan University Kritipur Nepal; ^3^ Department of Animal Nutrition and Fodder Production, Faculty of Animal Science, Veterinary Science and Fisheries (FAVF) Agriculture and Forestry University (AFU) Rampur Nepal; ^4^ Livestock Services Section, Guthichaur Rural Municipality Jumla Nepal; ^5^ Department of Animal Sciences, College of Food Agriculture and Environmental Sciences The Ohio State University Wooster Ohio USA; ^6^ Department of Poultry Science University of Georgia Athens Georgia USA

**Keywords:** broiler, coccidiosis, drinking water, *Eimeria*, probiotic, synbiotic

## Abstract

This study evaluated the effects of probiotic and synbiotic supplementation on growth performance, serum biochemistry, immune organ development, intestinal length and histomorphometry in broilers challenged with *Eimeria*. A total of 360 Cobb 500 broilers were assigned to six treatment groups (3 replicates of 20 birds), and from Day 14 to 35, birds received water supplemented with either probiotic, synbiotic or control. On Day 14, challenged groups received a 10× dose of *Eimeria* from a commercial live vaccine. Data were analyzed using a 2×3 factorial ANOVA. *Eimeria* challenge significantly reduced average weight gain, final body weight and feed conversion efficiency. Intestinal morphology was adversely affected, as evidenced by decreased villus height (VH) and villus height to crypt depth ratio (VCR) in both the duodenum and jejunum. Eimeria challenge also induced significant alterations in serum biochemical parameters, including alkaline phosphatase, albumin, urea, lipase and triglyceride levels. Probiotic and synbiotic supplementation significantly improved relative spleen weight, intestinal length (jejunum, ileum, cecum) and villus morphology in all intestinal segments. Significant main effects of treatment were observed for growth and serum enzymes. Interaction between coccidiosis infection and treatment was significant for urea, and duodenal VH and VCR. Overall, probiotic and synbiotic supplementation mitigated the adverse effects of coccidiosis, with no significant differences observed between the two interventions.

## Graphical abstract

This study assessed probiotic and synbiotic supplementation via drinking water in *Eimeria* challenged broilers. In a 2×3 factorial design with 360 Cobb 500 chickens, *Eimeria* impaired growth, FCR and gut morphology, while probiotics and synbiotics improved performance, intestinal structure and serum biochemistry, alleviating coccidiosis induced damage.

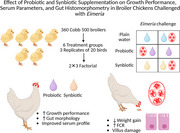

## 1 Introduction

Coccidiosis, a parasitic disease caused by protozoa of the genus *Eimeria*, poses a significant threat to the poultry industry worldwide. The disease's economic impact is evident through reduced growth rates, feed conversion rates and increased mortality among poultry (Chapman 2014; Abebe and Gugsa 2018). The economic loss due to coccidiosis is about USD 14 billion globally (Blake et al. 2020). The severity of coccidiosis varies with *Eimeria* species, dose and site of infection, ranging from mild enteritis with fluid loss and nutrient malabsorption *(E. acervulina*, *E. mitis)*, to intestinal inflammation with epithelial sloughing and haemorrhages (*E. brunett*i*, E. maxima*), and severe villus destruction with extensive haemorrhage and mortality (*E. necatrix*, *E. tenella*) (Chapman 2014). The clinical symptoms of coccidiosis in broilers are determined by the severity of the infection, which frequently depends upon the species of *Eimeria* (Abebe and Gugsa 2018). Some typical warning signs of coccidiosis are diarrhoea, dehydration, reduced feed intake, weight loss and elevated mortality (Chapman 2014; Gerhold et al. 2016). Control measures for coccidiosis primarily rely on the use of anticoccidial agents such as ionophores and chemicals like amprolium and sulfaquinoxaline (Chapman 2014). However, prolonged use of these drugs can lead to the development of drug‐resistant strains of *Eimeria*, posing a serious threat to flock health (Shirley and Lillehoj 2012). As a result, effective management strategies are necessary to combat this significant disease in poultry.

Probiotics and synbiotics are increasingly recognized as effective alternatives to conventional anticoccidial drugs in broiler production, particularly in developing poultry systems such as Nepal. These dietary supplements enhance intestinal health, reduce the severity of coccidiosis and improve growth performance, thereby decreasing reliance on antibiotics and chemical anticoccidials (Kabir 2009; Cheng et al. 2017; Prentza et al. 2022). The most commonly used probiotic strains in poultry are *Lactobacillus*, *Bifidobacterium* and *Bacillus* (Gaggìa et al. 2010). In addition, other strains such as *Enterococcus*, *Streptococcus* and *Escherichia coli* have also been used in poultry. Probiotic can improve gut health by competitive exclusion of pathogenic bacteria, reducing intestinal inflammation, enhancing gut barrier function and modulating the immune response (Knap et al. 2011). Synbiotics are combination of both probiotic and prebiotic and have emerged as a promising approach in animal nutrition regarding their potential benefits in poultry health, growth and productivity. Prebiotics serves as a selective feed ingredient for probiotic bacteria and exert synergistic beneficial effects in production and disease resilience in poultry. Commonly used synbiotics in poultry nutrition include combinations such as *Lactobacillus* with inulin, *Bifidobacterium* with oligofructose and *Streptococcus* with galactooligosaccharides (GOS). The synergistic effect of probiotics and prebiotics in synbiotics has been shown to enhance the survival and proliferation of beneficial gut microorganisms, leading to improved gut health, nutrient digestion and immune function (Rehman et al. 2020). In poultry production, probiotics and synbiotics supplementation has demonstrated benefits in improving growth performance, feed efficiency, gut health and reducing pathogen colonization (Pourabedin and Zhao 2015; Froebel et al. 2019; Zhang et al. 2021).

Supplementation with probiotics or synbiotics has consistently improved growth performance and feed efficiency in broilers. Synbiotic formulations containing *Lactobacillus acidophilus* with inulin, as well as probiotic mixtures including *L. acidophilus* and *Bacillus subtilis*, have been shown to increase body weight gain (BWG) and improve feed conversion ratio (FCR) (Awad et al. 2009). In addition to performance benefits, these supplements enhance immune competence, as indicated by increased relative weights of the thymus, spleen and bursa of Fabricius and elevated lymphocyte and macrophage populations in immune organs (Nikpiran et al. 2013; Fathi et al. 2017).

Probiotics and synbiotics also exert beneficial effects on metabolic and biochemical parameters. Reduced serum alanine aminotransferase (ALT) and aspartate aminotransferase (AST) concentrations have been reported following supplementation, reflecting improved hepatic function. In coccidiosis‐challenged broilers, additional reductions in serum urea nitrogen have been observed, suggesting alleviation of metabolic stress (Pourakbari et al. 2016; Wang et al. 2019). Furthermore, increases in serum total protein (TP) and albumin (ALB) levels have been consistently associated with probiotic and synbiotic supplementation, indicating improved protein metabolism and nutrient utilization (Dev et al. 2020; Yazhini et al. 2018).

Marked improvements in digestive function and intestinal morphology have also been documented. Probiotic and synbiotic supplementation enhance digestive enzyme activity, including amylase and lipase, particularly under coccidiosis challenge. These effects are accompanied by increased gut length, villus height (VH), and villus height to crypt depth ratio (VCR) in the small intestine, reflecting improved absorptive capacity and intestinal integrity (Awad et al. 2009; Calik et al. 2019; Mohamed et al. 2022).

Poultry industry is shifting from antibiotics to non‐antibiotic alternatives following concerns regarding antibiotic resistance. Optimizing productivity and health outcomes in the poultry industry necessitates the systematic evaluation of various interventions and the application of evidence‐based management practices. Innovative strategies are increasingly focused on enhancing the effectiveness of alternative approaches while minimizing the dependence on antibiotics in food‐producing animals (Khomayezi and Adewole 2022). Despite numerous studies, there are very less evidence on effectiveness of probiotics and synbiotics in Nepalese scenario. Therefore, this research aims to determine the impact of probiotic and synbiotic supplementation on growth performance, serum parameters and gut histomorphometry of broilers challenged with *Eimeria* spp. in hilly region of Nepal.

## 2 Materials and Methods

### 2.1 Study Site and Duration

The Animal Welfare Division, Ministry of Agriculture and Livestock Development, Bagmati Province, approved the animal care and use protocols for this study (Ref no: 1404). A licensed veterinarian in Nepal trained the research team in animal handling. Experiment was conducted at the poultry farm of Dhading Polytechnic School, Gajuri Rural Municipality‐1, Dhading, from March to June 2023. Laboratory work was conducted at Dhading Polytechnic School, serum biochemistry at the Veterinary Research and Diagnostic Laboratory (VRDL), and histomorphometry at the Center for Molecular Dynamics Nepal (CMDN), Kathmandu.

### 2.2 Birds, Experimental Treatments and Coccidiosis Infection

A total of 360 one‐day‐old Cobb 500 chicks were reared on deep litter in a circular cage system (10 m diameter) until Day 13. Birds were reared according to recommended Cobb 500 management practices, including maintenance of appropriate temperature, humidity, lighting regime, ventilation and stocking density (COBB Broiler Management Guide 2023). On Day 14, chicks were assigned to six treatment groups in a 2×3 factorial design: Two levels of *Eimeria* challenge (challenged and unchallenged) and three water treatments (plain water, probiotic, synbiotic) (Table 1). Each treatment was replicated in three pens with 20 birds per pen (5 sq. ft./bird). Feed and borewell water were provided ad libitum using commercial pelleted feed (Nepal Feed Ltd.): B0 (Day 0–14), B1 (Day 15–28), and B2 (Day 29–35) (Table 2).

**TABLE 1:** Different treatment used in current study.

		Water treatment		
		Plain Water	Probiotic	Synbiotic
***Eimeria* Challenge**	**Unchallenged**	Unchallenged + Plain water	Unchallenged + Probiotic	Unchallenged + Synbiotic
	**Challenged**	Challenged + Plain water	Challenged + Probiotic	Challenged + Synbiotic

**TABLE 2:** Nutritional Composition of feed used in current study.

Calculated nutrient composition	B0	B1	B2
Moisture	11	11	11
Crude protein	22	21	20
Crude fibre	6	6	6
Crude fat	3.5	3.5	3.5
Acid insoluble ash	3.5	3.5	3.5
Calcium	1	0.9	0.8
Salt as sodium chloride	0.5	0.5	0.5
Total phosphorus	0.7	0.7	0.7
Lysine	1.4	1.3	1.2
Methionine	0.5	0.5	0.5
Methionine and cysteine	1	0.9	0.9
Metabolizable energy kcal/kg	2900	3000	3100

On Day 14, birds in challenged groups were orally gavaged with 1 mL of a commercial live heptavalent *Eimeria* vaccine (Vaxxon Coccivet R, 10× recommended dose), while unchallenged groups received 1 mL of distilled water. The vaccine contained *E. acervulina*, *E. brunetti*, *E. maxima*, *E. necatrix*, *E. praecox*, *E. tenella*, and *E. mitis*, propagated in Specific Pathogen Free (SPF) birds. Post‐inoculation, birds were observed twice daily at 6‐h intervals. Probiotic (Yea‐Sacc, Alltech, KY, USA) and synbiotic (Provita, SOOCOM, China) were administered through drinking water from Day 14 at 1 g/L. The probiotic consisted of *Saccharomyces cerevisiae* strain 1026 (2.5 × 10^8^ CFU/g). The synbiotic included *Clostridium butyricum*, *B. subtilis*, *Enterococcus faecium*, *Lactobacillus* spp., and *bifidus* factor (5 × 10^8^ CFU/g). Probiotic and synbiotic supplementation were administered at a concentration of 1 g/L of drinking water. Daily water intake was monitored to ensure uniform availability of the supplemented water across treatment groups. The probiotic and synbiotic solutions were freshly prepared each day prior to administration to minimize loss of viability. Products were stored according to the manufacturer's recommendations, and supplementation was completed within the stated shelf life. Although formal stability testing under field conditions was not conducted, daily preparation was intended to maintain product potency and consistency throughout the experimental period.

### 2.3 Performance Parameters

The body weight (BW) was measured weekly starting from Day 14. Daily feed and water intake were recorded, along with mortality. Calculations were performed using standard formulas:

- Body weight gain (BWG): BWG was calculated as the difference between the average BW of two successive weeks.
  Weekly BWG (g) = Current week weight (g) − Previous week weight (g).
- Feed consumption (FC):
  Daily feed consumption (g) = Feed offered on previous day − Remaining feed.
  Weekly feed consumption (g) = Summation of daily feed consumption on 7 days.
- Feed conversion ratio (FCR):
  FCR = [Total feed consumption (g) at a particular age]/[Total live BW (g) at a particular age]
- Mortality: Treatment‐wise mortality percentage was calculated.
  Mortality Percentage = (Number of bird dead during experiment)/[Number of total birds per treatment (60)] × 100%

### 2.4 Gut Length and Organ Weight

On Day 35, ten birds per pen were euthanized. The gastrointestinal tract was dissected and divided into four regions: Duodenum (from ventriculus to bile duct), jejunum (from bile duct to Meckel's diverticulum), ileum (from Meckel's diverticulum to ileocecal junction), and cecum. The length of each section was measured. Relative gut length was calculated using: Relative Gut Length (cm/kg) = Length of gut segment (cm) / Live BW (kg). Weights of immune organs (spleen, bursa of fabricius, and thymus) were recorded on Days 21 and 35. Relative organ weight was calculated as: Relative Organ Weight (g/kg) = Organ weight (g) / Live BW (kg)

### 2.5 Biochemical Analysis

Blood samples were collected and serum was separated by centrifugation at 3000 rpm for 10 min and stored at −20°C until analysis. Serum biochemical parameters including AST (U/L), ALT (U/L), TP (g/dL), ALB (g/dL), urea (mg/dL), creatinine (mg/dL), amylase (mg/dL), lipase (U/L), total cholesterol (mg/dL) and triglycerides (TG, mg/dL) were analysed using **BioMaxima** clinical chemistry kits following the manufacturers’ instructions. Enzyme activities (AST, ALT, amylase, lipase) were determined using kinetic colorimetric methods, while TP, ALB, urea, creatinine, cholesterol and TG were measured using standard enzymatic colorimetric assays. All assays were run on a fully automated clinical chemistry analyzer such as the **Diatron Pictus 500** platform, with internal quality control procedures applied throughout the analytical process to ensure accuracy and reproducibility.

### 2.6 Sampling and Histological Slide Preparation

On Days 21 and 35, intestinal samples (duodenum, jejunum, ileum and cecum) were collected from 10 birds per group and fixed in 10% buffered formalin. Histological processing at Center of Molecular Dynamics (CMDN), Kathmandu, Nepal followed standard protocols: Formalin fixation, graded alcohol dehydration (70%, 90% and 95%), xylene clearing and paraffin embedding. Five‐micrometer tissue sections were cut using a rotary microtome, mounted on glass slides and stained with haematoxylin and eosin (H&E). Stained slides were air‐dried and prepared for microscopic evaluation.

### 2.7 Gut Histomorphometry

Histomorphometric measurements were conducted at the Pathology Laboratory, Polytechnic Institute, Chitwan, Nepal. VH and crypt depth (CD) were measured in duodenum, jejunum and ileum from ten well‐oriented villi per segment. VH was measured from tip to base, CD from the base of the villus to the base of the crypt. VCR was calculated. An Olympus AX70 microscope with a Sony DXC‐930P digital video camera was used for image analysis. Average VH, CD and VCR were computed per segment.

### 2.8 Statistical Analysis

All data were subjected to two‐way analysis of variance (ANOVA) using R statistical software version 4.2.2. The model evaluated the main effects of *Eimeria* challenge and water treatment, as well as their interaction. Differences among treatment means were determined using Duncan's Multiple Range Test (DMRT). Statistical significance was set at *p* < 0.05.

## 3 Results

### 3.1 Performance Parameters

The total feed intake (TFI), total water intake (TWI), total average weight gain (TAWG) and FCR from Day 14 to Day 35 of age, as influenced by *Eimeria* challenge and different water treatments is shown in Table 3. A significant interaction between *Eimeria* challenge and water treatment was observed for TFI (*p* < 0.001) and TWI (*p* < 0.001); however, no significant interaction was found for TAWG and FCR. In unchallenged birds, both probiotic and synbiotic supplementation reduced TFI and TWI compared to plain water. In challenged birds, probiotic supplementation reduced TFI compared to synbiotic and plain water treatments. Similarly, under challenge conditions, both probiotic and synbiotic supplementation increased TWI compared to plain water. In addition, both *Eimeria* challenge and water treatment had significant main effects on TAWG and FCR (*p* < 0.001). *Eimeria* challenge reduced TAWG and increased FCR. In contrast, both probiotic and synbiotic supplementation improved TAWG and reduced FCR, indicating beneficial effects on growth performance.

**TABLE 3:** Total feed intake (FI), total water intake (WI), total average weight gain (AWG) and feed conversion ratio (FCR) from Day 14 to Day 35 as influenced by *Eimeria* challenge and different water treatments on Cobb‐500 broilers.

Factors	Treatments	TFI (g)	TWI (mL)	TAWG (g)	FCR
*Interaction effect*					
Unchallenged × Plain		49130.00^a^	86045.00^a^	1548.43	1.67
Unchallenged × Synbiotic		46528.33^d^	84121.66^b^	1671.26	1.47
Unchallenged × Probiotic		46035.00^e^	82403.34^c^	1663.97	1.46
Challenged × Plain		48245.00^b^	80260.00^e^	1394.2	1.85
Challenged × Synbiotic		48235.00^b^	82760.00^c^	1523.13	1.68
Challenged × Probiotic		47308.33^c^	81176.66^d^	1511.6	1.67
	*p‐*value	<0.001	<0.001	0.952	0.177
	LSD (0.05)	314.08	789.10	31.19	0.026
SEM		42.313	103.26	3.98	0.004
CV %		0.36	0.49	1.1	0.88
Grand mean		47580.28	82794.44	1552.102	1.64
*Main effects*					
*Eimeria* challenge	Unchallenged	47231.11^b^	84190^a^	1627.89^a^	1.54^b^
	Challenged	47929.44^a^	81398.88^b^	1476.31^b^	1.74^a^
	*p‐*value	<0.001	<0.001	<0.001	<0.001
	LSD (0.05)	181.33	426.14	18.00	0.015
Water treatment	Plain	48687.5^a^	83152.5^a^	1471.31^b^	1.76^a^
	Synbiotic	47381.67^b^	83440.83^a^	1597.2^a^	1.58^b^
	Probiotic	46671.67^c^	81790^b^	1587.78^a^	1.56^b^
	*p‐*value	<0.001	<0.001	<0.001	<0.001
	LSD (0.05)	222.80	521.91	22.05	0.018

*Note*: Means with same letter in column are not significantly different at *p* = 0.05 by DMRT. (*p* < 0.05) = Significant at 5%, (*p* < 0.01) = significant at 1%.

Abbreviations: CV, Coefficient of variation; LSD, Least significant difference; SEM, Standard error of mean.

### 3.2 Body Weight Gain

Table 4 presents the BW of broilers on Days 21, 28 and 35 of age, as influenced by *Eimeria* challenge and different water treatments. There was significant interaction effect of *Eimeria* challenge and water treatments on BWG (*p* < 0.001) only at Day 21 of age. In both unchallenged and challenged birds, probiotic and synbiotic supplementation increased the BWG compared to plain water. Similarly, there was significant main effect of both *Eimeria* challenge and water treatment on BWG at Day 28 and Day 35 of age (*p* < 0.001). At Day 28 of age, *Eimeria* challenge decreased the BWG and probiotic and synbiotic supplementation increased the BWG of birds. Furthermore, at Day 35 of age, *Eimeria* challenge decreased the BWG and only synbiotic supplementation increased the BWG of birds.

**TABLE 4:** Body weight gain (BWG) at Day 21, Day 28 and Day 35 as influenced by *Eimeria* challenge and different water treatments in Cobb‐500 broilers.

Factors	Treatments	BWG at d21 (g)	BWG at d28 (g)	BWG at d35 (g)
*Interaction effect*				
Unchallenged × Plain		829.6^b^	1382.66	1971.46
Unchallenged × Synbiotic		893.46^a^	1489.53	2086.60
Unchallenged × Probiotic		888.20^a^	1485.00	2084.46
Challenged × Plain		730.60^d^	1284.467	1815.26
Challenged × Synbiotic		784.30^c^	1363.80	1934.40
Challenged × Probiotic		784.20^c^	1364.80	1930.66
	*p*‐value	<0.001	0.405	0.986
	LSD (0.05)	18.27	30.34	33.95
SEM		2.789	4.349	4.889
CV %		3.07	2.99	2.37
Grand mean		818.66	1395.04	1970.47
*Main Effects*				
*Eimeria* challenge	Unchallenged	870.42^a^	1452.40^a^	2047.51^a^
	Challenged	766.91^b^	1337.69^b^	1893.44^b^
	*p*‐value	<0.01	<0.001	<0.001
	LSD (0.05)	10.55	17.54	19.6
Water treatment	Plain	780.10^b^	1333.57^b^	1893.37^b^
	Synbiotic	838.833^a^	1426.67^a^	2010.50^a^
	Probiotic	837.06^a^	1424.9^a^	2007.56^b^
	*p*‐value	<0.001	<0.001	<0.001
	LSD (0.05)	12.92	21.45	24.00

*Note*: Means with same letter in column are not significantly different at *p* = 0.05 by DMRT. (*p *< 0.05) = Significant at 5%, (*p* < 0.01) = significant at 1%.

Abbreviations: CV, Coefficient of variation; LSD, Least significant difference; SEM, Standard error of mean.

### 3.3 Mortality

Highest mortality was seen in *Eimeria*‐challenged group administered with plain drinking water and lowest mortality was observed in the *Eimeria*‐unchallenged group administered with synbiotic.

### 3.4 Serum Parameters

Table 5 summarizes the serum biochemical parameters—AST, ALT, TP, ALB, urea and creatinine—on Day 35 of age as influenced by *Eimeria* challenge and water treatments. A significant interaction between *Eimeria* challenge and water treatment was observed for serum urea concentration (*p* = 0.01), while no significant interaction was noted for the other parameters. In unchallenged birds, neither probiotic nor synbiotic supplementation affected serum urea levels compared to plain water. However, in challenged birds, synbiotic supplementation significantly reduced serum urea concentration compared to plain water, while probiotic supplementation had no effect. In addition, water treatment exerted a significant main effect on serum AST levels (*p* = 0.01), with both probiotic and synbiotic supplementation resulting in reduced AST concentrations.

**TABLE 5:** Serum aspartate aminotransferase (AST), alanine aminotransferase (ALT), total protein (g/dL), albumin (ALB), urea and creatinine (Creat.) as influenced by *Eimeria* challenge and different water treatments on Cobb‐500 broilers.

Factors	Treatments	AST (U/L)	ALT (U/L)	TP (g/dL)	ALB (g/dL)	Urea (mg/dL)	Creat. (mg/dL)
*Combination effect*							
Unchallenged × Plain		154.54	18.68	4.826	2.77	6.94^d^	0.533
Unchallenged × Synbiotic		94.187	17.136	5.71	2.946	10.226^cd^	0.727
Unchallenged × Probiotic		107.07	27.18	5.76	2.91	9.736^cd^	0.707
Challenged × Plain		171.44	38.343	4.816	2.29	20.353^a^	0.73
Challenged × Synbiotic		107.37	27.743	5.27	2.74	12.576^bc^	0.793
Challenged × Probiotic		124.39	32.53	5.513	2.773	16.913^ab^	0.75
*p‐*value	0.993	0.601	0.836	0.499	0.01	0.697	
LSD (0.05)	58.83	23.96	1.16	0.50	4.45	0.33	
SEM	7.793	2.868	0.146	0.061	0.611	0.039	
CV %	25.56	48.89	12	10.07	19.14	25.51	
Grand mean	126.49	26.93	5.31	2.73	12.79	0.7	
*Main effects*							
*Eimeria* challenge	Unchallenged	118.6	20.998	5.432	2.875	8.967^b^	0.656
	Challenged	134.4	32.872	5.2	2.601	16.61^a^	0.758
	*p‐*value	0.32	0.061	0.441	0.044	<0.001	0.216
	LSD (0.05)	33.96	13.83	0.67	0.29	2.57	0.19
Water treatment	Plain	162.99^a^	28.511	4.821	2.53	13.646	0.632
	Synbiotic	100.78^b^	22.44	5.49	2.843	11.401	0.76
	Probiotic	115.73^b^	29.855	5.636	2.841	13.325	0.728
	*p‐*value	0.01	0.548	0.09	0.093	0.305	0.406
	LSD (0.05)	41.6	16.94	0.82	0.35	3.15	0.23

*Note*: Means with same letter in column are not significantly different at *p* = 0.05 by DMRT. (*p* < 0.05) = Significant at 5%, (*p* < 0.01) = significant at 1%.

Abbreviations: CV, Coefficient of variation; LSD, Least significant difference; SEM, Standard error of mean.

Table 6 presents the effects of *Eimeria* challenge and water treatments on serum levels of amylase, lipase, cholesterol and TG on Day 35. No significant interaction effects were observed for any of these parameters. However, *Eimeria* challenge significantly reduced serum lipase levels (*p* < 0.001). Water treatments had significant main effects on all four parameters (*p* < 0.001), where both probiotic and synbiotic supplementation increased serum amylase and lipase levels, and decreased cholesterol and TG concentrations.

**TABLE 6:** Serum amylase, lipase, cholesterol and triglycerides (TG) levels as influenced by *Eimeria* challenge and different water treatments on Cobb‐500 broilers.

Factors	Treatments	Amylase (mg/dL)	Lipase (U/L)	Cholesterol (mg/dL)	TG (mg/dL)
*Interaction effect*					
Unchallenged × Plain		161.926	26.31	147.553	97.75
Unchallenged × Synbiotic		390.713	35.156	111.803	78
Unchallenged × Probiotic		414.66	35.11	111.773	73.253
Challenged × Plain		153.846	22.456	155.513	111.796
Challenged × Synbiotic		377.613	29.703	124.066	76.813
Challenged × Probiotic		391.3	25.876	116.49	80.223
	*p‐*value	0.985	0.173	0.83	0.156
	LSD (0.05)	140.48	4.68	22.85	11.95
SEM		18.354	0.558	2.508	1.492
CV %		24.51	8.84	8.96	7.6
Grand mean		315.01	29.1	127.86	86.3
*Main Effects*					
*Eimeria* challenge	Unchallenged	322.433	32.192^a^	123.71	83.001
	Challenged	307.586	26.012^b^	132.023	89.611
	*p‐*value	0.693	<0.001	0.123	0.047
	LSD (0.05)	81.1	2.7	12.04	6.89
Water treatment	Plain	157.886^b^	24.383^b^	151.533^a^	104.773^a^
	Synbiotic	384.163^a^	32.43^a^	117.935^b^	77.406^b^
	Probiotic	402.98^a^	30.493^a^	114.131^b^	76.738^b^
	*p‐*value	<0.001	<0.001	<0.001	<0.001
	LSD (0.05)	99.33	3.31	14.74	8.44

*Note*: Means with same letter in column are not significantly different at *p* = 0.05 by DMRT. (*p* < 0.05) = Significant at 5%, (*p* < 0.01) = significant at 1%.

Abbreviations: CV, Coefficient of variation; LSD, Least significant difference; SEM, Standard error of mean.

### 3.5 Relative Gut Length

Table 7 presents the relative gut length measurements on Day 35 of age as influenced by *Eimeria* challenge and different water treatments. No significant interaction effect between *Eimeria* challenge and water treatment was observed for the relative lengths of the duodenum, jejunum, ileum, entire small intestine or cecum. However, *Eimeria* challenge exerted a significant main effect on the relative lengths of the duodenum (*p* = 0.003), jejunum (*p* = 0.03), and whole small intestine (*p* = 0.003), resulting in reduced lengths in challenged birds. Water treatment also had a significant main effect on the relative lengths of the jejunum (*p* = 0.007), ileum (*p* = 0.002), whole small intestine (*p* = 0.002) and cecum (*p* = 0.04). Both probiotic and synbiotic supplementation increased the relative lengths of the jejunum, ileum and whole small intestine compared to plain water. In addition, only probiotic supplementation increased the relative length of the cecum.

**TABLE 7:** Relative gut length as influenced by *Eimeria* challenge and different water treatments on Cobb‐500 broiler at Day 35.

Factors	Treatments	Duodenum (cm/kg)	Jejunum (cm/kg)	Ileum (cm/kg)	SI (cm/kg)	Cecum (cm/kg)
*Interaction effect*						
Unchallenged × Plain		19.226	38.926	38.383	96.536	19.833
Unchallenged × Synbiotic		19.883	40.816	40.64	101.34	20.213
Unchallenged × Probiotic		19.853	42.596	42.47	104.923	20.546
Challenged × Plain		17.346	38.496	37.73	93.573	19.326
Challenged × Synbiotic		18.76	39.963	39.76	98.483	19.776
Challenged × Probiotic		17.643	39.936	40.17	97.75	20.876
	*p‐*value	0.643	0.235	0.494	0.265	0.508
	LSD (0.05)	1.77	2.11	2.14	4.77	1.29
SEM		0.237	0.267	0.298	0.583	0.158
CV %		5.17	2.84	2.94	2.65	3.5
Grand mean		18.78	40.12	39.85	98.76	20.09
*Main effects*						
*Eimeria* challenge	Unchallenged	19.65^a^	40.78^a^	40.49	100.93^a^	20.19
	Challenged	17.916^b^	39.465^b^	39.22	96.60^b^	19.99
	*p‐*value	0.003	0.03	0.053	0.003	0.53
	LSD (0.05)	1.02	1.21	1.23	2.75	0.75
Water treatment	Plain	18.28	38.71^b^	38.05^b^	95.055^b^	19.58^b^
	Synbiotic	19.32	40.39^a^	40.2^a^	99.911^a^	19.995^b^
	Probiotic	18.75	41.26^a^	41.32^a^	101.336^a^	20.711^a^
	*p‐*value	0.244	0.007	0.002	0.002	0.038
	LSD (0.05)	1.25	1.49	1.5	3.37	0.92

*Note*: Means with same letter in column are not significantly different at *p* = 0.05 by DMRT. (*p* < 0.05) = Significant at 5%, (*p* < 0.01) = significant at 1%.

Abbreviations: CV, Coefficient of variation; LSD, Least significant difference; SEM, Standard error of mean; SI, Small intestine.

### 3.6 Relative Weight of Immune Organs

Table 8 presents the relative weights of immune organs (bursa, spleen and thymus) on Days 21 and 35 of age as influenced by *Eimeria* challenge and water treatments. No significant interaction effect between *Eimeria* challenge and water treatment was observed for the relative weights of any immune organ at either time point. However, *Eimeria* challenge exerted a significant main effect on the relative weights of the bursa (*p* < 0.001), spleen (*p* = 0.04) and thymus (*p* = 0.04) on Day 21. Birds exposed to the challenge showed reduced relative weights of all three immune organs compared to unchallenged birds. Water treatment also showed a significant main effect on spleen weight on both Day 21 (*p* = 0.01) and Day 35 (*p* = 0.02). At both time points, probiotic and synbiotic supplementation increased the relative spleen weight compared to plain water.

**TABLE 8:** Relative weight of bursa, spleen and thymus as influenced by *Eimeria* challenge and different water treatments on Cobb‐500 broilers.

		Day 21			Day 35		
Challenges	Treatments	Bursa (g/kg)	Spleen (g/kg)	Thymus (g/kg)	Bursa (g/kg)	Spleen (g/kg)	Thymus (g/kg)
*Interaction effect*							
Unchallenged × Plain		0.74	0.906	1.163	1.58	1.293	1.966
Unchallenged × Synbiotic		0.673	1.083	1.37	1.67	1.4	2.246
Unchallenged × Probiotic		0.72	1.136	1.206	1.65	1.396	2.26
Challenged × Plain		0.523	0.706	1.07	1.496	1.23	1.88
Challenged × Synbiotic		0.556	1.123	1.136	1.613	1.39	2.013
Challenged × Probiotic		0.566	0.84	1.153	1.593	1.376	2.053
	*p‐*value	0.42	0.141	0.39	0.942	0.829	0.748
	LSD (0.05)	0.115	0.25	0.2	0.14	0.14	0.31
SEM		0.019	0.031	0.025	0.019	0.018	0.039
CV %		10.1	8.2	9.66	4.84	5.89	8.45
Grand mean		20.09	0.966	1.18	1.6	1.34	2.07
*Main effects*							
*Eimeria* challenge	Unchallenged	0.71^a^	1.042	1.246^a^	1.633	1.363	2.157
	Challenged	0.548^b^	0.89	1.12^b^	1.567	1.332	1.982
	*p‐*value	<0.001	0.04	0.04	0.106	0.425	0.059
	LSD (0.05)	0.066	0.144	0.12	0.81	0.083	0.18
Water treatment	Plain	0.631	0.806^c^	1.116	1.538	1.261^b^	1.923
	Synbiotic	0.615	1.103^a^	1.253	1.641	1.395^a^	2.13
	Probiotic	0.643	0.988^b^	1.18	1.621	1.386^a^	2.156
	*p‐*value	0.747	0.011	0.16	0.097	0.02	0.084
	LSD (0.05)	0.082	0.177	0.147	0.09	0.1	0.22

*Note*: Means with same letter in column are not significantly different at *p* = 0.05 by DMRT. (*p* < 0.05) = Significant at 5%, (*p* < 0.01) = significant at 1%.

Abbreviations: CV, Coefficient of variation; LSD, Least significant difference; SEM, Standard error of mean.

### 3.7 Gut Histomorphometry

#### 3.7.1 Duodenum

Table 9 summarizes the histomorphometric measurements of the duodenum on Days 21 and 35 of age as influenced by *Eimeria* challenge and water treatments. A significant interaction between *Eimeria* challenge and water treatment was observed for VH (*p* = 0.003) and VCR (*p* = 0.003) on Day 21, and for VH (*p* < 0.001), CD (*p* = 0.002) and VCR (*p* = 0.01) on Day 35. At both time points, probiotic and synbiotic supplementation increased VH and VCR in both challenged and unchallenged birds compared to plain water. On Day 35, both supplements also increased CD in unchallenged birds, while only probiotic supplementation increased CD in challenged birds.

**TABLE 9:** Duodenal villus height (VH), crypt depth (CD), villus height: crypt depth ratio (VCR) as influenced by *Eimeria* challenge and different water treatments on Cobb‐500 broilers.

Factors	Treatments	Duodenum, D 21			Duodenum, D 35		
		VH (µm)	CD (µm)	VCR	VH (µm)	CD (µm)	VCR
*Interaction effect*							
Unchallenged × Plain		903.02^c^	216.5	4.32^d^	989.11^c^	231.7^b^	4.33^b^
Unchallenged × Synbiotic		1505.38^a^	185.08	8.50^a^	1629.82^a^	272.43^a^	5.99^a^
Unchallenged × Probiotic		1470.18^a^	214.31	7.07^b^	1633.56^a^	280.43^a^	6.06^a^
Challenged × Plain		778.86^d^	209.52	3.98^d^	878.45^d^	245.19^b^	3.62^c^
Challenged × Synbiotic		1145.50^b^	214.59	5.60^c^	1309.6^b^	231.03^b^	5.73^a^
Challenged × Probiotic		1171.79^b^	220.21	5.57^c^	1330.97^b^	291.61^a^	4.56^b^
	*p‐*value	0.003	0.33	0.003	<0.001	0.002	0.01
	LSD (0.05)	98.06	34.72	1.04	80.38	23.96	0.57
SEM		11.619	3.447	0.082	14.079	5.042	0.151
CV %		11.61	22.76	24.65	8.54	12.75	15.62
Grand mean		1162.45	210.03	5.84	1295.25	258.73	5.05
*Main effects*							
*Eimeria* challenge	Unchallenged	1292.86^a^	205.29	6.63^a^	1417.50^a^	261.51	5.46^a^
	Challenged	1032.05^b^	214.77	5.05^b^	1173.01^b^	255.94	4.63^b^
	*p‐*value	<0.001	0.35	<0.001	<0.001	0.424	<0.001
	LSD (0.05)	56.62	20.05	0.6	46.40	13.84	0.33
Water treatment	Plain	840.94^b^	213	4.15^b^	933.78^b^	238.44^b^	3.98^c^
	Synbiotic	1325.44^a^	199.84	7.05^a^	1469.7^a^	251.72^b^	5.86^a^
	Probiotic	1320.99^a^	217.26	6.32^a^	1482.26^a^	286.02^a^	5.31^b^
	*p‐*value	<0.001	0.344	<0.001	<0.001	<0.001	<0.001
	LSD (0.05)	69.34	24.56	0.74	56.83	23.96	0.57

*Note*: Means with same letter in column are not significantly different at *p* = 0.05 by DMRT. (*p* < 0.05) = Significant at 5%, (*p* < 0.01) = significant at 1%.

Abbreviations: CD, Crypt depth; CV, Coefficient of variation; LSD, Least significant difference; SEM, Standard error of mean; VCR, Villus height to crypt depth ratio; VH, Villus height.

#### 3.7.2 Jejunum

Table 10 presents the histomorphometric measurements of the jejunum on Days 21 and 35. A significant interaction effect of *Eimeria* challenge and water treatments was found for VH (*p* < 0.001) and VCR (*p* = 0.03) on Day 21. In unchallenged birds, both probiotic and synbiotic treatments increased VH and VCR, whereas in challenged birds, both supplements increased VH but not VCR. On Day 35, no significant interaction effect was observed for VH, CD or VCR. However, there was a significant main effect of *Eimeria* challenge on VH (*p* < 0.001), and significant main effects of water treatments on VH (*p* < 0.001) and VCR (*p* = 0.03). At this time point, coccidiosis challenge reduced VH, while both probiotic and synbiotic supplementation increased VH and VCR.

**TABLE 10:** Jejunal villus height (VH), crypt depth (CD), villus height: crypt depth ratio (VCR) as influenced by *Eimeria* challenge and different water treatments on Cobb‐500 broilers.

		Jejunum, D21			Jejunum, D35		
Challenges	Treatments	VH (µm)	CD (µm)	VCR	VH (µm)	CD (µm)	VCR
*Interaction effect*	
Unchallenged × Plain		733.7^e^	219.15	3.56^b^	897.42	205.07	4.6
Unchallenged × Synbiotic		919.49^a^	191.19	5.19^a^	1124.79	213.71	5.53
Unchallenged × Probiotic		887.96^b^	194.58	5.06^a^	1099.17	213.72	5.48
Challenged × Plain		763.13^e^	204.81	3.96^b^	868.2	190.38	4.75
Challenged × Synbiotic		807.80^d^	199.01	4.17^b^	1001.15	191.93	5.55
Challenged × Probiotic		840.03^c^	205.86	4.19^b^	1099.17	202.4	5.27
	*p‐*value	<0.001	0.597	0.038	0.154	0.912	0.876
	LSD (0.05)	29.79	37.93	0.84	67.97	34.98	8.85
SEM		9.747	5.029	0.141	4.404	5.478	0.122
CV %		4.96	25.79	26.54	9.32	23.73	25.86
Grand mean		825.35	202.43	4.36	1003.07	202.86	5.2
*Main effects*							
*Eimeria* challenge	Unchallenged	847.05^a^	201.64	4.60^a^	1040.46^a^	210.83	5.20
	Challenged	803.66^b^	203.33	4.11^b^	965.69^b^	194.9	5.19
	*p*‐value	<0.001	0.885	0.045	<0.001	0.12	0.679
	LSD (0.05)	17.19	21.89	0.48	39.24	23.74	5.1
Water treatment	Plain	748.41^b^	211.98	3.76^b^	882.81^b^	197.72	4.67^b^
	Synbiotic	863.65^a^	195.1	4.68^a^	1062.97^a^	202.82	5.54^a^
	Probiotic	863.99^a^	200.22	4.63^a^	1063.44^a^	208.05	5.38^a^
	*p*‐value	<0.001	0.442	0.003	<0.001	0.709	0.03
	LSD (0.05)	21.06	26.82	0.59	48.06	24.73	6.26

*Note*: Means with same letter in column are not significantly different at *p* = 0.05 by DMRT. (*p* < 0.05) = Significant at 5%, (*p* < 0.01) = Significant at 1%.

Abbreviations: CD, Crypt depth; CV, Coefficient of variation; LSD, Least significant difference; SEM, Standard error of mean; VCR, Villus height to crypt depth ratio; VH, Villus height.

#### 3.7.3 Ileum

Table 11 outlines the histomorphometric measurements of the ileum on Days 21 and 35. A significant interaction effect was detected for VH (*p* = 0.02) on Day 21. In unchallenged birds, probiotic supplementation increased VH, while in challenged birds, synbiotic supplementation resulted in higher VH compared to plain water. On Day 35, there were no significant interaction or main effects of *Eimeria* challenge or water treatments on VH, CD or VCR.

**TABLE 11:** Ileal villus height (VH), crypt depth (CD), villus height: crypt depth ratio (VCR) as influenced by *Eimeria* challenge and different water treatments on Cobb‐500 broilers.

		Ileum, D21			Ileum, D35		
Factors	Treatments	VH (µm)	CD (µm)	VCR	VH (µm)	CD (µm)	VCR
*Interaction effect*							
Unchallenged × Plain		653.48^cd^	168.62	4.09	835.88	244.34	3.84
Unchallenged × Synbiotic		693.57^abc^	194.1	3.97	850.27	250.07	3.84
Unchallenged × Probiotic		716.84^ab^	182.53	4.17	853.58	257.82	3.9
Challenged × Plain		637.33^d^	192.1	3.51	805.99	244.61	3.8
Challenged × Synbiotic		731.55^a^	187.4	4.1	894.57	233.44	4.38
Challenged × Probiotic		678.88^bcd^	191.27	3.76	846.65	247.66	3.99
	*p‐*value	0.02	0.468	0.464	0.267	0.758	0.313
	LSD (0.05)	39.5	34.29	0.85	65.25	26.58	0.56
SEM		9.433	3.584	0.082	5.760	4.943	0.122
CV %		7.93	25.37	29.59	10.59	15.45	19.39
Grand mean		685.27	186	3.93	847.82	219.04	3.96
*Main effects*							
*Eimeria* challenge	Unchallenged	687.96	181.75	4.08	846.58	223.91	3.86
	Challenged	682.59	190.26	3.79	849.07	214.18	4.06
	*p‐*value	0.641	0.395	0.245	0.896	0.178	0.229
	LSD (0.05)	22.81	19.79	0.49	37.67	14.19	0.32
Water treatment	Plain	645.40^b^	180.36	3.8	820.94	218.41	3.82
	Synbiotic	712.56^a^	190.75	4.03	872.42	217.11	4.11
	Probiotic	697.86^a^	186.9	3.97	850.12	221.61	3.95
	*p‐*value	<0.001	0.69	0.719	0.09	0.869	0.347
	LSD (0.05)	27.93	24.24	0.59	46.14	17.38	0.39

*Note*: Means with same letter in column are not significantly different at *p* = 0.05 by DMRT. (*p* < 0.05) = Significant at 5%, (*p* < 0.01) = significant at 1%.

Abbreviations: CD, Crypt depth; CV, Coefficient of variation; LSD, Least significant difference; SEM, Standard error of mean; VCR, Villus height to crypt depth ratio; VH, Villus height.

## 4 Discussion

The current study demonstrate that *Eimeria*‐challenged broilers exhibited elevated mortality. *Eimeria* infection causes severe intestinal damage which promotes colonization of pathogenic bacteria inviting secondary infection leading to mortality (Madlala et al. 2021). Water treatments with both probiotic and synbiotic application decreased the mortality in both challenged and unchallenged treatment groups. Probiotic and synbiotic supplementation could decreases mortality in *Eimeria*‐challenged chickens by enhancing immune function through increased antioxidant enzyme activity, reducing pathogenic *Clostridium* overgrowth, and improving intestinal barrier integrity via tight junction proteins and villus morphology (Duff et al. 2020; Madlala et al. 2021; Mohsin et al. 2022).

Water treatments with both probiotic and synbiotic decreased the TFI in both non‐challenged and challenged birds. Probiotic component increases the short chain fatty acids (SCFAs) production, which further stimulates the secretion of satiety hormone, as well as improve nutrient absorption, ultimately reducing the demand of feed intake (Shah et al. 2023). Similarly, coccidiosis typically leads to reduced feed intake due to intestinal damage and inflammation, and similar result was found by Reid and Pitois (1965). However, when probiotic and synbiotic was supplemented during challenge, the reduction of feed intake is relatively lesser than the untreated non‐infected birds. This relative improvement is due to probiotic bacteria helping in maintaining intestinal integrity and function, and improving nutrient absorption capacity, allowing birds to continue consuming adequate feed despite the challenge (Duff et al. 2020; Acharya et al. 2024). Both probiotic and synbiotic supplementation in drinking water decreased the TWI in non‐challenged birds, while increased the TWI in challenged birds. Probiotics reduce the water intake in non‐challenged broilers by enhancing nutrient absorption, stabilizing gut microbiota, improving intestinal barrier integrity, which together help to reduce the physiological need for excess drinking water (Awad et al. 2009; Mountzouris et al. 2010; Markowiak and Ślizewska 2018). In addition, prebiotic component of synbiotic may provide additional benefit by maintaining the osmotic balance in the intestinal environment, ultimately supporting the growth of beneficial bacteria (Guarino et al. 2020; Śliżewska et al. 2020). *Eimeria* challenge typically reduces the water intake, and similar result was found in another study (Reid and Pitois 1965). When probiotic and synbiotic was supplemented to challenged birds, there was relative increase in water intake because probiotic and synbiotic may reduce the severity of coccidial infection and associated intestinal damage. *Eimeria* challenge significantly decreased the TAWG and BWG of broiler birds, which was similar to the findings of Lee et al. (2020). However, water treatments with both probiotic and synbiotic significantly increased the TAWG and BWG, and similar results was observed in other studies (Duff et al. 2020; Acharya et al. 2024). This improved wt. gain might be due to reduced intestinal damage, modulation of inflammatory response and direct inhibition of parasite reproduction by probiotic and synbiotic, reducing overall challenge burden (Duff et al. 2020; Acharya et al. 2024).

The FCR is critical performance and economic parameter in broiler production which is negatively impacted during coccidial infections, and similar finding was seen in our experiment too. However, both probiotic and synbiotic supplementation in water improved the FCR, and similar findings was observed by other studies (Midilli et al. 2008; Mookiah et al. 2014). Both probiotic and synbiotic supplementation improve the FCR by enhancing the nutrient availability, digestibility and absorption, reducing the intestinal inflammatory response and improving the gut barrier function (Śliżewska et al. 2020; Idowu et al. 2025).

Bood serum urea and AST are critical indicator of protein utilization and liver damage respectively in broilers. Synbiotic supplementation during *Eimeria* challenge significantly reduced the serum urea level, which suggests the improved protein utilization and absorption, by promoting beneficial gut microbes that utilize ammonia, reducing its conversion to urea in liver (LatipuDin et al. 2024; Liu et al. 2025). Similarly, both probiotic and synbiotic supplementation decreased the serum AST level irrespective of challenge, which might be due to reduced liver stress and damage. Both probiotic and synbiotic supplementation strengthen the gut barrier and limits the translocation of endotoxins to the liver, which prevents the hepatic inflammation and injury (Liu et al. 2025), and their antioxidant effects neutralize free radicals to protect liver cells from oxidative damage, leading to reduction of AST in blood serum (LatipuDin et al. 2024).

Lipase and amylase are digestive enzymes that hydrolyze dietary fats and starch, respectively into simpler forms for optimum absorption and metabolic utilization. *Eimeria* challenge significantly reduced the serum amylase level because *Eimeria* damages the gut lining and disrupts pancreatic function. In addition, intestinal lesions and reduced feed intake can leads in endogenous enzymes insufficiency in challenged birds (Kiarie et al. 2019). However, probiotic and synbiotic supplementation through water significantly decreased the serum amylase and lipase levels in birds, and similar finding was observed in a study by Mohamed et al. (2022). Probiotic bacteria can elevate the lipase and amylase levels by secreting their one enzymes (lipases and amylases) as well as stimulating intestinal and pancreatic cells to increase secretion of endogenous enzymes (Mohamed et al. 2022; Wang et al. 2024).

Serum cholesterol and TG are blood lipids essential for cellular functions and energy storage, but elevated levels can indicate dysregulated lipid metabolism and increased disease risk. Both probiotic and synbiotic supplementation through water significantly decreased the serum cholesterol and TG levels in broilers, and similar findings was observed in previous studies (Yazhini et al. 2018; Mohamed et al. 2022). Several probiotic bacteria including *Lactobacillus* and *Bifidobacterium*, binds cholesterol to their cell walls and efficiently remove it from the intestinal lumen. Some probiotic bacteria often produce the enzyme bile salt hydrolase, which deconjugates the bile acids, reduce the reabsorption of deconjugated bile acids and increase the excretion rate of cholesterol (since cholesterol is used to synthesize new bile acids).

Relative gut length is a critical indicator of gut development and capacity for nutrient absorption. *Eimeria* challenge significantly reduced the relative length of different segments of the intestine (duodenum, jejunum and entire SI). This might be due to extensive mucosal damage which is caused by *Eimeria* parasites during cycles of replication that physically destroys enterocytes and villi, leading to lesions and haemorrhages in the gut wall, ultimately resulting in morphological alterations of the intestinal mucosa and reduction of absorptive surface area (Nabian et al. 2018). This further leads to poor growth and reduced wt. gain and increased FCR (Cowieson et al. 2020), resulting in under‐developed and may be shorter and lighter intestinal segments. In addition, severe inflammation can cause fibrosis or thickening of intestinal tissue (Graham et al. 2023), which might reduce its elasticity and extensibility, potentially shortening the effective length of the gut. Conversely, both probiotic and synbiotic supplementation through water increase the relative length of different segments of the intestine (jejunum, ileum, entire SI and cecum), and similar finding was observed in a study (Stęczny and Kokoszyński 2020). Both probiotic and prebiotic supplementation decreases the colonization of pathogenic bacteria in gut, reduce the intestinal inflammation and increase the production of short chained fatty acid (SCFA) in hind‐gut (Wang et al. 2024), that improves the intestinal epithelial cells and help thicken and lengthen the gut lining.

Bursa of Fabricius and thymus are primary lymphoid organs in poultry, crucial for B cell and T cell development respectively, and their relative weights reflect immune status of the chickens. Our study indicated that *Eimeria* challenge significantly decreased the relative wt. of bursa of Fabricius and thymus, and similar findings was observed in multiple studies (Awadalla 1988; Li et al. 2009; Gottardo et al. 2017). This might be due to an immunological stress caused by coccidial infection. During severe coccidial infection, there is marked elevation of corticosteroids (stress hormones), which can induce the rapid atrophy of lymphoid organs such as bursa of Fabricius and thymus (Dohms and Metz 1991). In addition, coccidiosis may directly damage the gut‐associated lymphoid tissues and disrupt the immune cell production, further contributing to reduced relative wt. of bursa of Fabricius and thymus. Furthermore, spleen is a secondary lymphoid organs that filters blood and modulate immune responses by producing lymphocytes and antibodies. An active, healthy immune system may have moderately enlarged spleen due to higher lymphocyte proliferation. In our study, both probiotic and synbiotic supplementation increased the relative wt. of spleen, and similar finding was observed in previous studies (Li et al. 2009; Hashemitabar and Hosseinian 2024). This might be because several components of both probiotic and prebiotic act as immunostimulants, and can stimulate gut‐associated lymphoid tissue, which in turn signals peripheral lymphoid organs to develop (Li et al. 2009), and improves the relative wt. of spleen.

VH and CD are critical histomorphometric parameters of gut health, where taller villi provide more absorptive surface, while an optimal CD indicates healthy tissue turnover. A higher VCR generally signifies more efficient nutrient absorption. In our study, we found both probiotic and synbiotic supplementation increased either VH, CD, and VCR or all irrespective of the *Eimeria* challenge, and similar findings was observed in multiple studies (Hashemitabar and Hosseinian 2024; Wang et al. 2024). Both probiotic and prebiotic components induce the production of SCFAs which serve as a fuel for enterocytes as well as triggers the release of trophic hormones like GLP‐2, which expands the VH and CD (Hashemitabar and Hosseinian 2024). Probiotic bacteria also suppress pathogenic bacteria that could deteriorate gut lining (Gopal and Dhanasekaran 2021) as well as reduce gut inflammatory response by increased production of anti‐inflammatory cytokines (IL‐10), and reduction of pro‐inflammatory cytokines (Shah et al. 2023). In addition, amylase also induce the growth of villi (Ritz et al. 1995), and our study suggested the increase amylase activity in probiotic and synbiotic supplemented groups, which might be contributing the increased length of villi, crypts and VCR in probiotic and synbiotic supplemented treatment groups.

## 5 Conclusions

The present study highlights the beneficial effects of both probiotic and synbiotic supplementation in mitigating the adverse impacts of *Eimeria* infection in broiler chickens. *Eimeria*‐challenged birds exhibited increased mortality, impaired growth performance, compromised gut health and altered physiological and immune parameters. However, water‐based administration of probiotics and synbiotics significantly alleviated these effects by enhancing intestinal morphology, strengthening gut barrier integrity, improving nutrient absorption, modulating digestive enzyme activities and supporting immune organ development. In addition, supplementation reduced serum biomarkers of liver damage and lipid imbalance, further demonstrating systemic health benefits. These findings suggest that strategic supplementation with probiotic or synbiotic products via drinking water can serve as an effective, non‐antibiotic approach to improve broiler performance and resilience under *Eimeria* challenge, supporting sustainable poultry production. However, further research is warranted to assess their long‐term efficacy and economic feasibility under commercial production conditions.

## Author Contributions

**Subash Chhetri**: writing – original draft, writing – review and editing, conceptualization, data curation, formal analysis, investigation, methodology, resources, visualization, software. **Dinesh Kumar Singh**: writing – review and editing, resources, validation, supervision. **Bibash Bahadur Tiwari**: writing – review and editing, methodology. **Sushil Neupane**: writing – reveiw & editing, methodology. **Bikas Raj Shah**: writing – review and editing, formal analysis, resources, supervision, validation, visualization. **Deepak Subedi**: writing – review and editing, formal analysis, resources, visualization.

## Funding

The authors have nothing to report.

## Ethics Statement

The study was approved by the Animal Welfare Division, Ministry of Agriculture and Livestock Development, Bagmati Province, Nepal (Reference Number: 1404, approval date: 2,February 2023).

## Consent

The authors have nothing to report.

## Conflicts of Interest

The authors declare no conflicts of interest.

## Data Availability

The original contributions presented in this study are included in the article. Further inquiries can be directed to the corresponding author.
